# Psychological treatments for depression and anxiety in dementia and mild cognitive impairment: systematic review and meta-analysis^†^

**DOI:** 10.1192/bjp.bp.114.148130

**Published:** 2015-10

**Authors:** Vasiliki Orgeta, Afifa Qazi, Aimee Spector, Martin Orrell

**Affiliations:** **Vasiliki Orgeta**, PhD, Division of Psychiatry, University College London; **Afifa Qazi**, MBBS, MRCPsych, Goodmayes Hospital, North East London Foundation Trust; **Aimee Spector**, PhD, DClinPsych, Research Department of Clinical, Educational and Health Psychology, University College London; **Martin Orrell**, PhD, Institute of Mental Health, Nottingham, UK

## Abstract

**Background**

Anxiety and depression are common in people with dementia and mild cognitive impairment (MCI), but there is uncertainty about the effectiveness of both pharmacological and psychological therapies.

**Aims**

To evaluate the evidence of effectiveness of psychological treatments in treating depression and anxiety in people with dementia and MCI.

**Method**

We carried out a systematic review and meta-analysis of randomised controlled trials (RCTs) of psychological treatment versus usual care in people with dementia and MCI. Primary outcomes were symptoms of anxiety and depression. Secondary outcomes were quality of life, ability to perform daily activities, neuropsychiatric symptoms, cognition and caregivers' self-rated depressive symptoms.

**Results**

We included six RCTs, involving 439 participants with dementia, which used cognitive–behavioural therapy, interpersonal therapy, counselling or multimodal interventions including a specific psychological therapy. We found beneficial effects for both depression and anxiety. Overall, the quality of the evidence was moderate for depression and low for anxiety, due to the methodological limitations of the studies we identified and the limited number of trials.

**Conclusions**

The evidence from six RCTs suggests that psychological treatments are effective in reducing symptoms of depression and anxiety for people with dementia. There is a need for high-quality, multicentre trials including standardised, well-defined interventions.

Depression and anxiety are common in people with dementia and mild cognitive impairment (MCI). Estimates of prevalence of depressive symptoms in people with dementia range between 10 and 62%,^1^ with substantially lower rates when employing strict criteria for major depression.^2^ People with MCI are also susceptible to depression, with rates reported as moderate at 36%^3^ to high at 63%.^4^ Anxiety symptoms are equally frequent, if not more prevalent, with rates between 8 and 71%^5^ for people with dementia and between 10 and 74%^6^ for people with MCI. Relatively less is known about prevalence of anxiety disorders in dementia and MCI, with rates up to 49% for anxiety-specific disorders^7^ when using the Consortium to Establish a Registry for Alzheimer's Disease Behavioral Rating Scale for Dementia (CERAD-BRSD) criteria.^8^

Anxiety and depression have a substantial impact on outcomes as they decrease the ability to live independently,^9,10^ increase the risk of institutionalisation^11,12^ and result in higher caregiver burden.^13,14^ In people with MCI, early symptoms of depression can often be resistant to antidepressants,^15^ whereas both depression and anxiety have been found to predict higher rates of progression to Alzheimer's disease.^16^

Recent recommendations have stressed that the treatment of anxiety and depressive symptoms should be an essential part of the treatment of Alzheimer's disease and other dementias.^17^ Although pharmacological approaches are commonly used for anxiety and depression in dementia, these can have side-effects and remain largely ineffective.^18^ Further limitations include only a small number of trials conducted to date with small sample sizes,^19^ with most studies investigating classes of antidepressants not used routinely in treating depression in people with dementia in clinical practice.^20,21^ Therefore, psychological treatments adapted for use with people with cognitive impairment may offer an alternative approach. Other reviews have concluded that psychotherapy reduces depression in older adults with depressive symptoms^22^ and that psychological treatments can increase general psychological well-being in late-life depressive disorders.^23^ There are no reviews of studies, however, evaluating psychological treatments in people with dementia and MCI. In contrast to previous reviews, the present review focuses on psychological interventions for people with dementia or MCI, defined as any psychotherapeutic approach aimed at treating depression and anxiety, according to the World Health Organization criteria, such as cognitive–behavioural therapy (CBT), psychodynamic therapy, interpersonal therapy and supportive counselling.^24^

So, in comparison to previous reviews evaluating interventions that target anxiety and depression by incorporating some psychological elements (e.g. reminiscence^25^), or focusing on environmental changes^26^ or exercise,^27^ the primary objective of this review was to determine whether psychological interventions reduce depression and anxiety in people with dementia and MCI. Secondary objectives were to assess whether: (a) psychological interventions improve patient quality of life, cognition, activities of daily living (ADL), and reduce behavioural and psychological symptoms of dementia other than anxiety and depression compared with usual care; and (b) whether psychological treatments improve caregiver quality of life or reduce caregiver burden. This article is based on a Cochrane Review by the same authors, with full details of the review published by the Cochrane Library.^28^

## Method

We searched the Cochrane Dementia and Cognitive Improvement Group's Specialized Register and major healthcare databases, such as MEDLINE, Embase, CINHAL, PsycINFO, ALIOS and LILACS; our search also included a number of grey literature sources. Database searching was completed in January 2013. We searched for all ‘Treatment MCI’ and ‘Treatment Dementia’ studies with additional relevant terms. To view a list of all sources searched for the ALOIS database, see the ALOIS website (www.medicine.ox.ac.uk/alois/). We searched the identified citations for additional trials and contacted the corresponding authors of the identified trials for additional references and unpublished data. We scanned the reference lists of the identified publications and all review papers that were related to depression and anxiety in dementia and MCI.

We included all randomised controlled trials (RCTs) that included a control group (usual care) or comparison group receiving no specific psychological intervention. Additional criteria were that the study provided adequate information about study design and results, and separate data on participants with dementia or MCI. Inclusion criteria for participants were older adults diagnosed with dementia, Alzheimer's disease or organic brain syndrome, according to the DSM-IV, ICD-10 or comparable, and participants with a diagnosis of MCI, in any setting (e.g. home, community, institution). Any definition of MCI was acceptable as long as the criteria used were published and included evidence of objective cognitive impairment but no dementia.^29–31^

In this review, we considered any psychological therapy designed to reduce depressive and anxiety symptoms in people with dementia, which was defined as any intervention that: (a) was designed to reduce anxiety and depression or improve adaptive functioning; (b) was based on a psychological theory; and (c) involved a structured interaction between a facilitator and a participant, incorporating psychological methods. Eligible interventions included: (a) CBTs (which include CBT, cognitive analytic therapy, behavioural therapy or behaviour management therapy, brief rational insight and problem-solving therapy); (b) relaxation training therapies (e.g. progressive muscle relaxation); (c) psychodynamic therapies (including brief psychotherapy and insight-orientated psychotherapy); (d) interpersonal therapies; and (e) supportive/counselling therapies. We excluded treatments identified as medication, exercise, reminiscence therapy, music therapy, art and drama therapy, befriending or bibliotherapy.

Control conditions included no treatment (usual care) or a comparison group engaging in non-specific psychosocial activity (e.g. attention control, controlling for effects of staff attention or social contact). We did not consider comparisons with other therapeutic interventions in this review. We included studies that used combinations of different psychological treatments or combinations of pharmacological and psychological interventions. Primary outcomes were depression and anxiety, including clinician, caregiver and self-ratings. Secondary outcomes were patient quality of life, cognition, daily activity level (e.g. ADL), frequency of neuropsychiatric symptoms (e.g. Neuropsychiatric Inventory, NPI), and caregivers' quality of life or experience of caregiver burden.

Two reviewers (V.O., A.Q.) worked independently to identify RCTs that met the inclusion criteria, and extracted data independently. They discussed any disagreements with the fourth (M.O.) and third author (A.S.). We contacted the authors of the primary trials if there were doubts regarding missing data or the methodological details of the trial. We employed the approach recommended by the Cochrane Handbook for Systematic Reviews of Interventions (Cochrane Collaboration, Oxford, UK; see http://handbook.cochrane.org/), for assessing risk of bias, addressing the domains of sequence generation, allocation concealment, masking, incomplete outcome data, selective reporting and other issues. We used a fixed-effects model to represent overall estimate effects. We used standardised mean differences in some of the analyses as not all studies used the same outcome scale. We conducted all calculations with RevMan 5.0 for Windows (Cochrane Collaboration, Oxford, UK; see http://tech.cochrane.org/revman/download). We assessed heterogeneity between the included studies with the chi-squared test.^32^ We considered *P*-values <0.10 to be statistically significant. We quantified heterogeneity by using the *I*^2^ statistic.

## Results

**Fig. 1:**
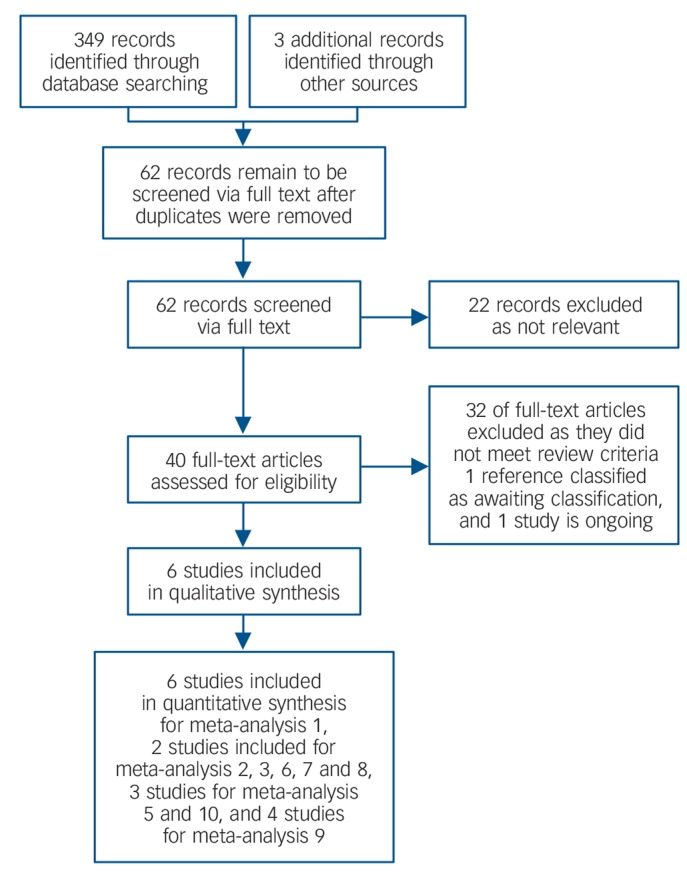
Review and meta-analysis flow diagram.

We identified a total of 349 references through database searching (January 2013), with three additional references identified (i.e. reference lists of identified studies and reviews of the literature). After removal of duplicates and clearly irrelevant articles, we retrieved 62 full text records. Of these 62 references, we could exclude 22 at this stage as not relevant, leaving 40 full text references to be fully assessed for eligibility. Of these, we excluded a total of 32 studies as they did not meet the review criteria, one study is ongoing and one study is awaiting classification, with further information required to clarify whether it would meet the inclusion criteria of this review. Thus, we found six studies to be eligible for inclusion. See Fig. 1 for details of the search process.

### Description of studies

The main study characteristics are shown in online Table DS1. We were able to pool data for depression from all six studies.^33–38^ When testing the effects of psychological treatment on anxiety only two studies contributed data. We pooled data from three and two studies for self-rated and caregiver-rated quality of life respectively. We pooled data from two studies to test the effects of psychological treatment on ADL and on the effects on neuropsychiatric symptoms. We pooled four studies for analyses on the effects on cognition. No evidence of heterogeneity was detected in the pooled studies, using the chi-squared test. The final analysis included pooled data from three studies on the effects of caregiver depression. In this analysis heterogeneity was evident.

### Primary outcomes

#### Depression

**Fig. 2:**
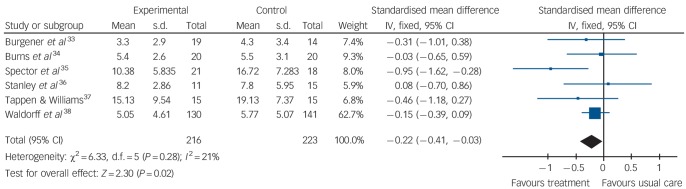
Forest plot of psychological treatment versus treatment as usual. Outcome: 1.1 Depression.

The first meta-analysis on the effects of psychological treatment on depression included 439 participants. Results significantly favoured psychological treatment (6 studies, standardised mean difference (SMD) −0.22; 95% CI −0.41 to −0.03) in reducing depressive symptoms for people with dementia (Fig. 2), with little heterogeneity between studies (*I*^2^ = 21%).

#### Anxiety

**Fig. 3:**
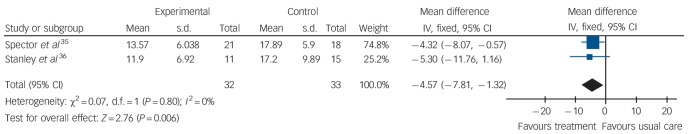
Forest plot of psychological treatment versus treatment as usual. Outcome: 1.2 Anxiety RAID. RAID, Rating Anxiety in Dementia scale.

**Fig. 4:**
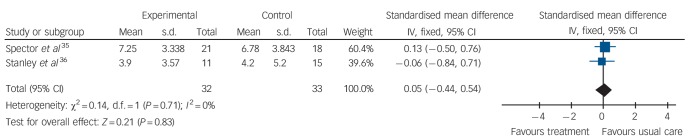
Forest plot of psychological treatment versus treatment as usual. Outcome: 1.3 Anxiety (self-ratings).

**Fig. 5:**
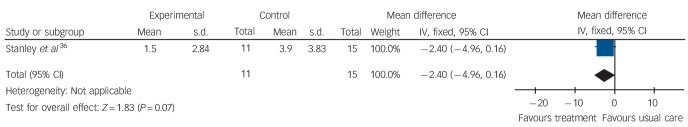
Forest plot of psychological treatment versus treatment as usual. Outcome: 1.4 Anxiety NPI-A. NPI-A, Neuropsychiatric Inventory-Anxiety.

Psychological treatment reduced clinician-rated anxiety measured with the Rating Anxiety in Dementia scale (Fig. 3) (2 studies, 65 participants, mean difference (MD) −4.57; 95% CI −7.81 to −1.32). However, there was no effect on self-rated anxiety (Fig. 4) (2 studies, 65 participants, SMD 0.05; 95% CI −0.44 to 0.54) or caregiver-rated anxiety measured with the NPI-Anxiety (Fig. 5) (1 study, 26 participants, MD −2.40; 95% CI −4.96 to 0.16).

### Secondary outcomes

#### Quality of life

Psychological treatment had no effect on patient self-rated quality of life (online Fig. DS1) (3 studies, 334 participants, MD 0.37; 95% CI −1.01 to 1.75) or on caregiver-rated patient quality of life (online Fig. DS2) (2 studies, 313 participants, MD 0.66; 95% CI −0.77 to 2.09).

#### Activities of daily living

Psychological treatment had no effect on ADL for people with dementia (online Fig. DS3) (2 studies, 313 participants, SMD −0.13; 95% CI −0.35 to 0.09).

#### Neuropsychiatric symptoms

Psychological treatment had no effect on neuropsychiatric symptoms (online Fig. DS4) (2 studies, 311 participants, SMD 0.06; 95% CI −0.16 to 0.28).

#### Cognition

Psychological treatment had no effect on cognition (online Fig. DS5) (4 studies, 381 participants, MD −0.80; 95% CI −1.70 to 0.11).

#### Caregiver depression

Psychological treatment for people with dementia had no effect on caregivers' depressive symptoms (online Fig. DS6) (3 studies, 337 participants, SMD 0.07; 95% CI −0.14 to 0.29), with moderate heterogeneity between studies in this analysis.

### Adverse events

None of the studies reported or described any adverse events.

## Discussion

The results of six RCTs with a total of 439 participants (216 receiving psychological treatment, 223 in control groups) showed that psychological treatments reduce depressive symptoms in people with dementia. Data from two studies showed that psychological treatments benefit people with dementia by reducing anxiety symptoms measured with a clinician-rated tool. These results compare favourably with recent studies, which found minimal or no benefits of pharmacological interventions in treating depression in dementia.^39^ Although both anxiety and depression were primary outcomes for this review, only two suitable studies included data for anxiety, and there was no effect of psychological treatment on secondary outcomes, such as ADL, quality of life, neuropsychiatric symptoms and cognition, or on caregiver depression.

The psychological therapies considered in this review stem from various theoretical perspectives, and in all studies individual protocols described the therapies in detail. Moreover, all studies targeted symptoms of anxiety and depression through a structured psychological approach (therapist and patient communication), which included directly teaching people with dementia skills to reduce anxiety and depression. Nevertheless, the trials we have included in this review evaluated a range of different psychological interventions and some used a combination of treatments. The length and duration of intervention also varied in the studies, leading to differences in intensity and frequency of the psychological treatment. A limitation of this review, therefore, is the substantial variation between studies in terms of the nature, duration and intensity of the psychological therapy evaluated, which may contribute to difficulties when interpreting the data.

No trials of psychological treatment aimed at people with MCI met our inclusion criteria. The three studies identified either did not employ an RCT design, participants had a cognitive impairment that was not specified according to the established criteria of MCI or the intervention that was evaluated was psychologically based but specifically targeted cognitive decline. None of the studies included reported adverse events.

### Quality of the evidence

Risk of bias was unclear for multiple domains in a large proportion of the studies, with the information provided by the published reports proving insufficient to determine the risk of bias associated with key methodological indicators. We classified only one of the studies as low risk in all domains of the Cochrane Collaboration's tool for assessing risk of bias. We classified the remaining five studies as being at unclear risk of bias in certain domains, due to limitations such as uncertainties about random sequence generation, allocation concealment, masking of participants and personnel, and outcome assessment. There was also evidence of selective reporting in one trial. Based on the Grades of Recommendation, Assessment, Development and Evaluation (GRADE) system, we have classified the quality of the evidence as ‘moderate’ for depression and ‘low’ for anxiety, due to the methodological limitations and the limited number of trials.

### Overall completeness and applicability of evidence

The studies we included in the present review only partially answered the research questions we posed. Few studies provided data on secondary symptoms of anxiety and we could not perform any subgroup analyses. Most studies to date have been conducted in the USA and Europe, limiting generalisability to the rest of the world. In most studies, information on concurrent psychotropic treatments was limited. Most participants had mild dementia, but one trial was conducted with nursing home residents who had more severe dementia.

The review followed guidelines set out by the Cochrane Collaboration.^40^ We used a comprehensive and sensitive strategy to identify studies; the first (V.O.) and second author (A.Q.) independently conducted the selection of studies, data extraction and assessments of risk of bias. The present review presents and discusses all outcomes described in the protocol that were available for analysis, regardless of whether or not there was statistical significance. Finally, it is worth noting that there were differences in terms of acceptance into treatment protocols (for example, the requirement of criteria for anxiety in two trials), which is likely to have resulted in some studies that were overly inclusive and others that exercised more conservative guidelines.

Results showed that psychological treatments are superior to usual care in reducing depression and anxiety, although we were not able to investigate whether they are superior to active controls. However, in some studies the control condition was enriched beyond usual care, as opposed to standard care, indicating that the efficacy of psychological therapies may be potentially underestimated.

The current review is distinctive in systematically analysing psychological interventions to reduce anxiety or depression that are conducted primarily with people with dementia, rather than focusing on environmental changes or skills building for family caregivers.^41^ Previous reviews have concentrated on the effectiveness of other interventions of a psychosocial nature (including cognitive stimulation, cognitive rehabilitation, reminiscence and activity-based interventions), which are not aimed specifically at anxiety or depression.^42,43^ These reviews do suggest that non-pharmacological interventions can be useful, and potentially cost-effective, in terms of improving psychological outcomes.^42,43^ However, for some studies there was a lack of clear psychiatric diagnosis of depression or anxiety, or a low baseline level of anxiety and depressive symptoms. These factors limit interpretation of the results, so it is uncertain how far these findings may be applicable for people with a specific diagnosis of anxiety or depression, or with higher baseline levels of depression and anxiety.

### Implications for practice and research

There is moderate quality evidence that psychological treatments can reduce depressive symptoms in people with dementia, and limited evidence that they can reduce anxiety. Although the effect size for depression was small, the beneficial effects for anxiety suggest that psychological approaches may be associated with potential improvements in both depressive and anxiety symptoms. The findings of this review compare favourably with limited evidence base on pharmacological treatment for anxiety and current evidence of weak support for the use of antidepressants when treating depression in dementia.^39^ Considering that there were no adverse events reported related to the use of psychological treatments, we can conclude that the observed effects are of meaningful clinical benefit to people with dementia.

The small number of studies in this review and the variations in the type and duration of treatment make it difficult to draw conclusions about the best way to provide psychological treatment. There is a need for well-designed, multicentre RCTs that adhere to the high standards of methodology and reporting, following the Consolidated Standards of Reporting Trials (CONSORT) statement. These trials should focus on standardised theory-based psychological therapies, rather than multimodal approaches which combine a variety of approaches. The lack of follow-up data makes it difficult to use this research to inform evidence-based policy about how best to deliver psychological therapy services, and this is compounded by the lack of data about cost-effectiveness. Future studies should also examine the longer-term effects of psychological treatment for people with dementia.
